# Establishing a cardiac training group for patients with heart failure: the “HIP-in-Würzburg” study

**DOI:** 10.1007/s00392-021-01892-1

**Published:** 2021-06-22

**Authors:** Gülmisal Güder, Joana Wilkesmann, Nina Scholz, Robert Leppich, Peter Düking, Billy Sperlich, Christian Rost, Stefan Frantz, Caroline Morbach, Floran Sahiti, Ulrich Stefenelli, Margret Breunig, Stefan Störk

**Affiliations:** 1 Department of Internal Medicine I, University Hospital Würzburg, CardiologyWürzburg, Germany; 2 Comprehensive Heart Failure Center, University and University Hospital Würzburg, Würzburg, Germany; 3 Department of Computer Science, Software Engineering Group, University of Würzburg, Würzburg, Germany; 4 Department of Sport Science, Integrative and Experimental Exercise Science, University of Würzburg, Würzburg, Germany; 5 Practice for Cardiology, Mainherz, Würzburg, Germany; 6 Joint Practice for Nephrology and Cardiology, Niere Mit Herz, Wertheim, Germany

**Keywords:** Heart failure, Cardiac training group, Heart failure training group, m exercise training

## Abstract

**Background:**

Exercise training in heart failure (HF) is recommended but not routinely offered, because of logistic and safety-related reasons. In 2020, the German Society for Prevention&Rehabilitation and the German Society for Cardiology requested establishing dedicated “HF training groups.” Here, we aimed to implement and evaluate the feasibility and safety of one of the first HF training groups in Germany.

**Methods:**

Twelve patients (three women) with symptomatic HF (NYHA class II/III) and an ejection fraction ≤ 45% participated and were offered weekly, physician-supervised exercise training for 1 year. Patients received a wrist-worn pedometer (M430 Polar) and underwent the following assessments at baseline and after 4, 8 and 12 months: cardiopulmonary exercise test, 6-min walk test, echocardiography (blinded reading), and quality of life assessment (Kansas City Cardiomyopathy Questionnaire, KCCQ).

**Results:**

All patients (median age [quartiles] 64 [49; 64] years) completed the study and participated in 76% of the offered 36 training sessions. The pedometer was worn ≥ 1000 min per day over 86% of the time. No cardiovascular events occurred during training. Across 12 months, NT-proBNP dropped from 986 pg/ml [455; 1937] to 483 pg/ml [247; 2322], and LVEF increased from 36% [29;41] to 41% [32;46]%, (*p* for trend = 0.01). We observed no changes in exercise capacity except for a subtle increase in peak VO_2_% predicted, from 66.5 [49; 77] to 67 [52; 78]; p for trend = 0.03. The physical function and social limitation domains of the KCCQ improved from 60 [54; 82] to 71 [58; 95, and from 63 [39; 83] to 78 [64; 92]; p for trend = 0.04 and = 0.01, respectively. Positive trends were further seen for the clinical and overall summary scores.

**Conclusion:**

This pilot study showed that the implementation of a supervised HF-exercise program is feasible, safe, and has the potential to improve both quality of life and surrogate markers of HF severity. This first exercise experiment should facilitate the design of risk-adopted training programs for patients with HF.

## Introduction

In Germany, the concept of ambulatory “cardiac training groups” (HSGs, *Herzsportgruppen*) supervised by a physician with emergency equipment and an experienced exercise physiologist was initiated and implemented as early as 1965. The HSGs aimed to safely enable patients with cardiac diseases to regain their neuromuscular and cardiovascular performance after a cardiac event, predominantly after a myocardial infarction [1]. Since then, more than 6,000 HSGs have been founded in Germany offering structured exercise training available on prescription [2].

Despite well-established evidence and guidelines recommending physical activity to improve health-related quality of life and to reduce hospitalization rates in patients with heart failure, their participation in the above-mentioned HSGs is heavily underrepresented, most likely because of their perceived higher risk for cardiovascular events during training sessions [3–5]. However, multiple studies over the last three decades consistently reported that the risk of events like sudden cardiac death, cardiac arrest, or myocardial infarction in exercising patients with cardiovascular diseases was small, and progressively diminished over the years [6]. Nevertheless, heart failure patients carry a higher baseline risk, which might be particularly increased for unfit or previously sedentary patients performing uncontrolled or even vigorous (un-)supervised training [7]. Therefore, and complementary to established HSGs, the German Society for Prevention and Rehabilitation and the German Society for Cardiology in 2020 claimed the establishment of dedicated “Heart Failure Training Groups” for patients with heart failure and limited exercise capacity, who cannot participate in conventional HSGs [6, 8].

The current prospective pilot study aimed to scientifically follow one of the very first heart failure training groups founded in Germany, examine its feasibility and safety, and investigate its effects on exercise capacity and quality of life in the time course of one year.

## Methods

### Study population

Between November 2018 and May 2019, 12 ambulatory patients with chronic heart failure were enrolled into the “HIP-in-Würzburg” pilot study, a non-randomized, monocentric, prospective cohort study. Adult patients were eligible if they had symptomatic heart failure (New York Heart Association functional class II or III) with a reduced left ventricular ejection fraction (LVEF) below 45%, and were physically and mentally capable of and willing to participate in a weekly training program. The study was conducted according to the Good Clinical Practice guidelines and the Declaration of Helsinki principles of 2002, and was approved by the responsible ethic committee of Würzburg, Germany (221/18-sc). All patients provided written informed consent.

### Patient assessment

***Study flow.*** All patients underwent a standardized clinical evaluation including assessment of medical history, quality of life (Kansas City Cardiomyopathy Questionnaire; KCCQ), and depressed mood (Patient Health Questionnaire; PHQ-9). Venous blood was sampled for routine cardiovascular markers. Additional investigations included a 12-lead resting electrocardiogram (ECG), standardized echocardiography, a 6-min walk test (6MWT), and a cardiopulmonary exercise test (CPET). All investigations were performed in identical fashion by trained staff at baseline and after 4, 8, and 12 months of follow-up.

#### Questionnaires

Both questionnaires were completed by the patients themselves, i.e., without interference of relatives or staff, at baseline and at each follow-up visit. Depressive symptoms were assessed by the validated German version of the PHQ-9 questionnaire that consists of nine questions, each one indexed with 0 to 3 points [9]. A total PHQ-9 sum score of 10 or above suggests the presence of depression [10]. Disease-specific quality of life was assessed by the Kansas City Cardiomyopathy Questionnaire (KCCQ), a 23-item, self-administered instrument that quantifies six domains (physical limitation, symptom frequency and stability, self-efficacy, social limitation, and quality of life) [11]. The items are further aggregated to the sum-scales “functional status” (physical limitation, symptom frequency and stability) and overall summary score (all items except self-efficacy and symptom stability). The scores are transformed to a 0-to-100-point scale, where lower scores represent more severe symptoms or limitations and scores of 100 indicate no symptoms, no limitations, and excellent quality of life [11, 12].

***Echocardiography*** was performed according to practice guidelines by a certified echo technician [13] and focused on the following measurements: left ventricular systolic function (LVEF, assessed either by the biplane-modified Simpson method or visually by eye balling in the apical four-chamber view, in cases of poor ultrasound condition), left atrial diameter measured at the end-systole (LADs), left ventricular end-diastolic diameter (LVEDD), septal and posterior wall diameter, diastolic dysfunction (ratio E/A, E/e’, deceleration time), and presence of valvular disease. The LVEF measurements were re-assessed by an independent certified sonographer (FS), who was blinded to patient name, history, and follow-up time point.

#### Six-minute walk test (6MWT)

The 6MWT measures the distance a patient covers walking quickly on a flat, hard surface for 6 min. This submaximal exercise test was first validated in heart failure in 1985, and shows a very good correlation with NYHA functional class, bicycle ergometry and other parameters of physical functioning in patients with heart failure [14, 15]. The test was performed by experienced staff on a dedicated 20-m walkway in outpatient clinics under standardized conditions.

#### Cardiopulmonary exercise testing (CPET)

Serial spiroergometry was performed on the treadmill or bicycle ergometer according to the preference of the patient, and the following standard parameters were assessed: heart rate, blood pressure, minute ventilation (VE), respiratory exchange ratio (RER), maximal oxygen consumption (peakVO_2_) respiratory equivalent for oxygen (EqO_2_) and carbon dioxide (EqCO_2_). The type of CPET exercise procedure remained constant during the course of the study. A ramp protocol was used and gradually increased until physical exhaustion or achievement of termination criteria suggested by current guidelines [16].Before and immediately after exercise testing, capillary blood gas analysis was performed with assessment of standard parameters: pH, lactate, base excess, partial pressure of O_2_ (pO_2_) and CO_2_ (pCO_2_).

### Training program and activity monitor

#### Group sessions

Patients met once per week as an additional group, i.e., separate from patients participating in the conventional cardiac training program. Before each training session, blood pressure, pulse and body mass were assessed in each patient. A certified HSG instructor offered an individualized training program to each participant with exercise intensity based on information derived from the baseline CPET. Patients were equipped with a heart rate monitor and activity tracker (Polar M430, Polar Electro Oy, Kempele, Finland), and were encouraged to adjust their training intensity according to the target heart rate (determined by means of CPET at 70% of peakVO_2_ ± 10 beats per minute). The information derived from the follow-up CPET was applied to adjust the target heart rate. In general, a moderate training intensity (11–13 on Borg’s 6–20 scale) was targeted [17]. Each training session lasted 60 min. The aim of each session was to improve muscular strength, endurance and coordination, as well as self-perception. The training consisted of the modules described in Table 1.

**Table 1:** Description of content of training sessions

Time (minutes)	Exercise type	Description of a typical training session
	Pre-exercise measurements	Measuring weight, pulse, and blood pressure
5	Welcoming & warm-up	Walking in circles, variation of step sequences
20	Strength training	Training with Thera Band and low-weight dumb-bells (0.5–1 kg; 3 × 10 repetitions), avoidance of deep breathings
15	Endurance training	Depending on the fitness level of the patient: walking, sit-and-walk, steeplechase Training intensity controlled by heart rate and ratings of perceived exertion (Borg scale)
10	Strength, balance & coordination training	Walking and standing; using balls, batons and hoops of different sizes; partner training
10	Relaxation & cool-down	Lying on mat; listening to music; additional breathing exercises
	Closing	Pulse measurement

Intensity of each training unit was adapted to the individual exercise capacity of the patient

#### Activity monitor

Daily step counts were collected to monitor physical activity behavior during waking hours. As patients had to get used to wearing and using the watch, the first interval for step count monitoring covered four weeks (“run-in-phase,” and patients were followed up at month four (FUP4), eight (FUP8) and 12 (FUP12). The analysis comprise of the data consisting for at least 600 min between 06:00 and 22:00 per day, and 1000 min per 24-h period in total.

#### Training at home

All patients were iteratively encouraged to increase their daily activity level, i.e., to observe their daily step count and also to exercise on days with no group meetings. The supervising physician set individual goals for home training, e.g., a 10% increase in the daily step. Depending on the patient’s success, further increase or reduction was advised to ensure motivation. Patients walking more than 10,000 steps per day were advised to include other exercising activities such as jogging, walking, or bike riding with heart rate control. Patients feeling unwell were advised to stop training at home. Each patient received weekly reports of the daily step count and activity level.

### Clinical endpoints

The primary endpoint was defined as the 12-month change in peakVO_2_ at CPET. Secondary endpoints evaluated the safety of the training program (frequency of cardiovascular events as heart failure hospitalization and cardiovascular deaths during follow-up), changes over time in the distance walked during the 6MWT, as well as domains of quality of life, and improvements in echocardiographic and laboratory markers of heart failure. The incidence of cardiovascular events as heart failure hospitalization and cardiovascular deaths was also assessed. Owing to the pilot character of the study, no formal sample size calculation and power analysis were performed.

### Data analysis

Continuous variables are reported as mean (standard deviation) or median (quartiles), and categorical variables as absolute and relative frequencies. Trend tests for continuous variables from a single sample were calculated with Page´s rank trend test in R [18]. For binary variables, two analyses were combined [18, 19]. First, for each individual the slope was calculated from the course of the binary data over time. For instance, the slope was regarded as positive/negative if the values increased (e.g., 0–1-1–1 or 0–0-1–1 etc.) or decreased (e.g., 1–1-1–0; 1–1-0–0 etc.) over time. If the values remained unaltered over time, the slope was zero. Then, a one-sample Wilcoxon rank test was performed, to ascertain whether slopes differed from zero. As trend tests require complete data sets, missing values (up to 1 per variable) were imputed through missing value analysis with linear interpolation [20]. For binary variables, the last value was carried forward. All p-values are two-sided and are considered exploratory by nature.

## Results

### Study population and adherence to study procedures

A total of 12 patients were recruited for this pilot study. Their characteristics are summarized in Table 2, and those of the entire group are in Table 3. The median age of the participants was 64 years (49; 73), 25% were female, 33% had ischemic cardiomyopathy, 59% were in NYHA functional class III, and 75% exhibited heart failure with reduced ejection fraction. At study start, 83% received pharmacotherapy fully compliant with current guidelines [3]. At each study visit, heart failure medication was re-checked and optimized whenever possible and all completed the study after 12 months. Only one patient had missed one follow-up visit (8-month visit), otherwise, follow-up was complete. In total, 36 training sessions could be offered within 12 months, and the average participation rate was 76% (range 49–92%). The last recruited patient (pt. #12) was only offered 30 training units, because of the COVID-19 pandemic-induced lockdown in Germany in March 2020.

**Table 2:** Baseline characteristics of each patient

Patient no	Age, years	Sex	Cause of heart failure	NYHA class	LVEF, %	BMI, kg/m^2^	peakVO_2_, kg/ml/min	6MWT distance, m
#1	42	Male	DCM	II	33	29.9	25.5	500
#2	77	Male	Non-specific	II	34	22.0	18.4	460
#3	74	Male	ICM	III	28	29.7	9.0	320
#4	70	Male	ICM	III	35	32.1	11.0	320
#5	65	Male	Non-specific	II	41	30.1	17.8	520
#6	63	Male	DCM	II	45	30.2	17.3	440
#7	72	Female	ICM	III	44	26.8	10.3	440
#8	48	Male	DCM	III	20	33.6	10.7	380
#9	77	Female	DCM	III	41	26.8	16.5	440
#10	60	Male	ICM	III	37	32.8	11.5	480
#11	38	Male	Valvular	II	21	19.1	14.0	520
#12	53	Female	DCM	III	40	28.1	14.0	520

**Table 3:** Baseline characteristics of the study sample

Characteristic	*N* = 12
Male sex	9 (75)
Age (years)	64 (49; 64)
Body mass index (kg/m^2^)	29.8 (26.8; 31.6)
Duration of heart failure > 5 years	9 (75)
Ischemic cause of heart failure	4 (33.3)
NYHA class III	7 (58.3)
LVEF, %	36 (29; 41)
ICD/CRT	6 (50%)
Diabetes mellitus	3 (25)
Chronic obstructive lung disease (%)	3 (25)
Hemoglobin, g/dl	13.2(12.7; 13.9)
eGFR, ml/min/1.73m^2^	62 (53; 77)
NT-proBNP, pg/ml	985 (455; 1936)
Medication	
Optimal medical therapy*	10 (83)
ACEi/ARB (%)	6 (50)
ARNI (%)	5 (42)
Betablocker (%)	10 (83)
Mineralocorticoid receptor antagonist (%)	8 (67)
Diuretics (%)	6 (50)
Ivabradine (%)	2 (17)

Data are *n* (%) or median (quartiles)

*According to ESC heart failure guidelines 2016

LVEF, left ventricular ejection fraction; NYHA, New York Heart Association class; ICD, implantable cardioverter defibrillator; CRT-(D), cardiac resynchronization therapy (defibrillator); eGFR, estimated glomerular filtration rate [37]; NT-proBNP, amino-terminal pro-brain natriuretic peptide; ACEi/ARB, angiotensin converting enzyme inhibitor or angiotensin receptor blocker; ARNI, angiotensin receptor neprilysin inhibitor

### Safety

During training sessions, no cardiac or any other adverse events occurred. During 12 months of follow-up, three patients (pts. #4, #7, #11) were hospitalized due to causes unrelated to training. Patient #4 experienced ventricular tachycardia/fibrillation and was shocked multiple times by his implanted cardioverter-defibrillator (ICD). An acute coronary syndrome could be excluded, but coronary angiography revealed progression of his underlying multi-vessel coronary artery disease. Patient #7 was hospitalized twice, once to exclude progression of his coronary artery disease and once for tachyarrhythmia, necessitating electrical cardioversion. Patient #9 was also hospitalized twice, once for implantation of a subcutaneous ICD and once for cardiac decompensation. Of note, 12 months prior to inclusion into the study, five out of the 12 patients had been hospitalized for various reasons: pt. #1 twice, first for elective right heart catheterization and then for cardiac decompensation; pt. #2 twice, for myocardial biopsy and for exclusion of acute coronary syndrome; pt. #4 for angina pectoris necessitating stent implantation; pt. #8 was admitted six times in total, three times for cardiac decompensation, once for right heart catheterization, once for MitraClip® implantation, and once for transplant evaluation; pt. #12 was admitted for heart transplant evaluation.

### Pharmacotherapy of heart failure, biomarkers, and surrogates of heart failure severity

At baseline, 10 out of 12 patients received optimal medical therapy (OMT) according to the then-current ESC guidelines [3]. During follow-up, heart failure medication was further optimized: In one patient, beta-blocker was started and well tolerated; in three patients, ACEi/ARB changed to angiotensin receptor neprilysin inhibitor (ARNI), thus at the 12-month follow-up, eight patients were receiving ARNI (p for trend = 0.17). At the end of the study, all patients were on OMT (p for trend = 0.35; Table 4).

**Table 4:** Changes in heart failure medication and lipid lowering therapy during follow-up

	Baseline	Month 4	Month 8	Month 12	*P* for trend*
	*N* = 12	*N* = 12	*N* = 11	*N* = 12	
ACEi /ARB, n (%)	6 (50)	5 (42)	6 (55)	4 (33)	1.0
ARNI, n (%)	5 (42)	7 (58)	5 (45)	8 (67)	0.19
Betablocker, n (%)	10 (83)	10 (83)	9 (82)	11 (92)	1.0
MRA, n (%)	8 (67)	8 (67)	7 (64)	8 (67)	N.A
Ivabradine, n (%)	2 (17)	3 (25)	3 (27)	3 (25)	1.0
Diuretics, n (%)	6 (50)	7 (58)	5 (45)	6 (50)	0.56
Lipid lowering drug, n (%)	5 (42)	5 (42)	5 (45)	5 (42)	N.A
OMT, n (%)	10 (83)	12 (100)	11 (100)	12 (100)	0.35

*Page’s linear trend test. *N.A.* Page test not applicable

ACEi/ARB, angiotensin converting enzyme inhibitor or angiotensin receptor blocker; ARNI, angiotensin receptor neprilysin inhibitor, MRA mineralocorticoid receptor blocker, OMT optimal medical therapy

**Fig. 1:**
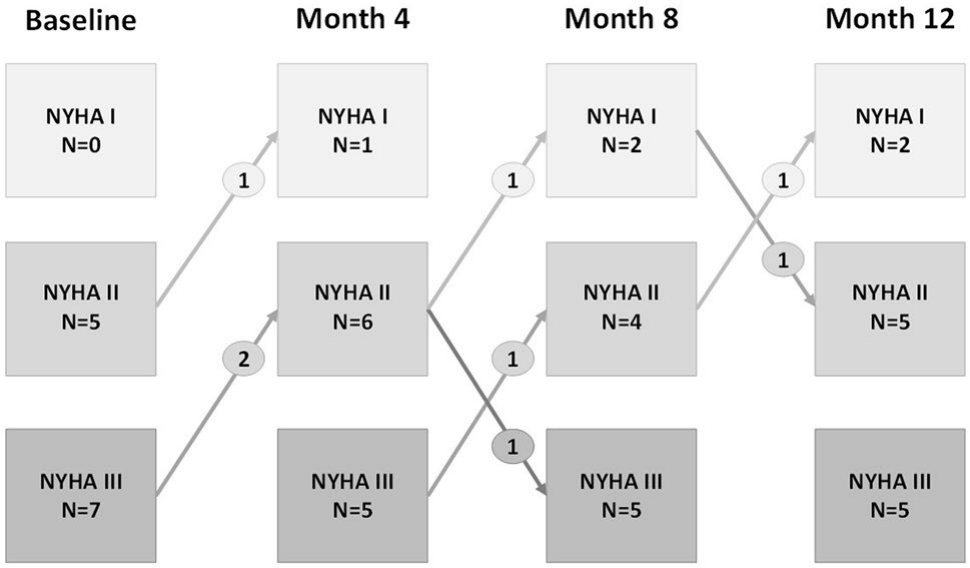
Changes in NYHA functional classes over the 12-month study period (n = 12). NYHA New York Heart association functional class

Despite no apparent changes in lipid lowering medication, total cholesterol and LDL cholesterol decreased from baseline to the end of the study (p for trend = 0.01 and 0.02; Table 5), whereas HDL cholesterol remained unaffected. Changes in NYHA functional class were nominally favorable but yielded no significant trend (Fig. 1 and Table 5). Subtle, yet significant improvements were further observed during follow-up for NT-proBNP levels (p for trend 0.01), and LVEF (p for trend < 0.01),

**Table 5:** Changes during follow-up

	Baseline N = 12	4 Months N = 12	8 Months N = 11	12 Months N = 12	P for trend*
Indicators of heart failure and metabolic changes					
NYHA I + II/III, n	5/7	7/5	6/5	7/5	0.33
LVEF (%)	36 (29; 41)	39 (31; 42)	41 (36; 48)	41 (32; 46)	** < 0.01**
NT-proBNP, pg/ml	986 (455; 1937)	676 (379; 1119)	477 (301; 1847)	483 (247; 2322)	**0.01**
Total cholesterol, mg /dl	176 (153; 226)	175 (140; 200)	165 (135; 197)	168 (153; 202)	**0.01**
LDL cholesterol, mg/dl	95 (82; 128)	93 (68; 116)	89 (66; 107)	88 (73; 110)	**0.02**
HDL cholesterol, mg/dl	54 (45; 59)	50 (45; 57)	52 (41; 59)	53 (44; 59)	0.13
***PHQ-9 sum score***	5.5 (2.3; 9.5)	5.5 (2; 11)	6 (2; 9)	5.5 (1.5; 10)	0.37
KCCQ domains & scores					
Symptoms	79 (65; 92)	86 (68; 95)	90 (73; 95)	84 (66; 92)	0.28
Physical function	60 (54; 82)	67 (51; 91)	75 (58; 90)	71 (58; 95)	**0.04**
Quality of Life	62.5 (35; 81)	75 (46; 94)	67 (50; 83)	67 (38; 98)	0.31
Social limitation	63 (39; 83)	78 (39; 91)	69 (50; 94)	78 (64; 92)	**0.01**
Self-efficacy	88 (75;100)	88 (75;100)	100 (75; 100)	94 (66; 100)	0.16
Symptom stability	50 (50; 50)	50 (50; 69)	50 (50; 50)	50 (50; 50)	0.27
Clinical summary score	67 (60; 86	77 (61; 93)	82 (68; 91)	77 (61; 96)	**0.05**
Overall summary score	62 (50; 84)	82 (51; 87)	74 (59; 92)	73 (57; 92)	0.09
Exercise capacity					
PeakVO_2_, ml/min/kg	14 (10.8; 17.7)	15 (11.7; 17.5)	15 (13; 17.8)	13.5 (12.2; 15.5)	**0.03**
PeakVO_2_, % of predicted	66.5 (49; 77)	78 (53; 83)	79 (54; 84)	67 (52; 78)	**0.03**
VO_2_ at VT1, ml/kg/min	9.6 (8.2; 12.12)	9.15 (8.4; 10.6)	10.5 (7.7; 11.3)	10.8 (8.9; 13.5)	0.19
VO_2_ at VT2, ml/kg/min	12.9 (9.8; 16.8)	13.4 (11.1; 16.9)	13.5 (10.9; 16.4)	11.9 (10.9; 15.6)	0.19
Heart rate at max exercise, bpm	108 (98; 132)	101 (96; 134)	102 (89; 120)	112 (93; 129)	0.10
Work rate at max exercise, W	86 (64; 111) ^+^	87 (72; 105) ^+^	91 (60; 115) ^#^	85 (56; 111) ^+^	0.23
Work rate at max exercise, % of predicted, W	73.5 (42; 98) ^$^	77.5 (40.5; 104) ^§^	88 (60; 102) ^#^	81.5 (46; 99) ^+^	0.25
6MWT distance, m	450 (395; 515)	465 (390; 520)	460 (400; 520)	470 (405; 530)	0.11
Step count, steps/day	10,566 (4590; 12,434)	9979 (5185; 13,614)	9412 (5787; 12,064)	9383 (5449; 11,095)	0.11
Activity time, min/day	477 (384; 524)	478 (359; 543)	470 (364; 575)	487 (337;547)	0.22

*Page´s linear trend test. *p*-value < 0.05 is considered as significant trend

VT, ventilatory threshold; 6MWT, 6-min walk test; LVEF, left ventricular ejection fraction; NYHA, New York Heart Association; KCCQ, Kansas City Cardiomyopathy Questionnaire; PHQ, Patient Health Questionnaire

Values are median (quartiles), unless indicated otherwise

^§^*N* = 9; ^#^*N* = 8

### Quality of life and mood

The KCCQ clinical and overall summary score both had improved nominally after 12 months (p for trend = 0.05 and = 0.09, respectively: Table 5). A significant improvement in physical function and social limitation was observed across the 12-month period (p for trend = 0.04 and = 0.01, respectively). By contrast, all other subscales were not materially altered over time (Table 5). Depressive mood, as assessed by the PHQ-9 summary score, was unchanged during follow-up.

### Exercise capacity

**Figure 2:**
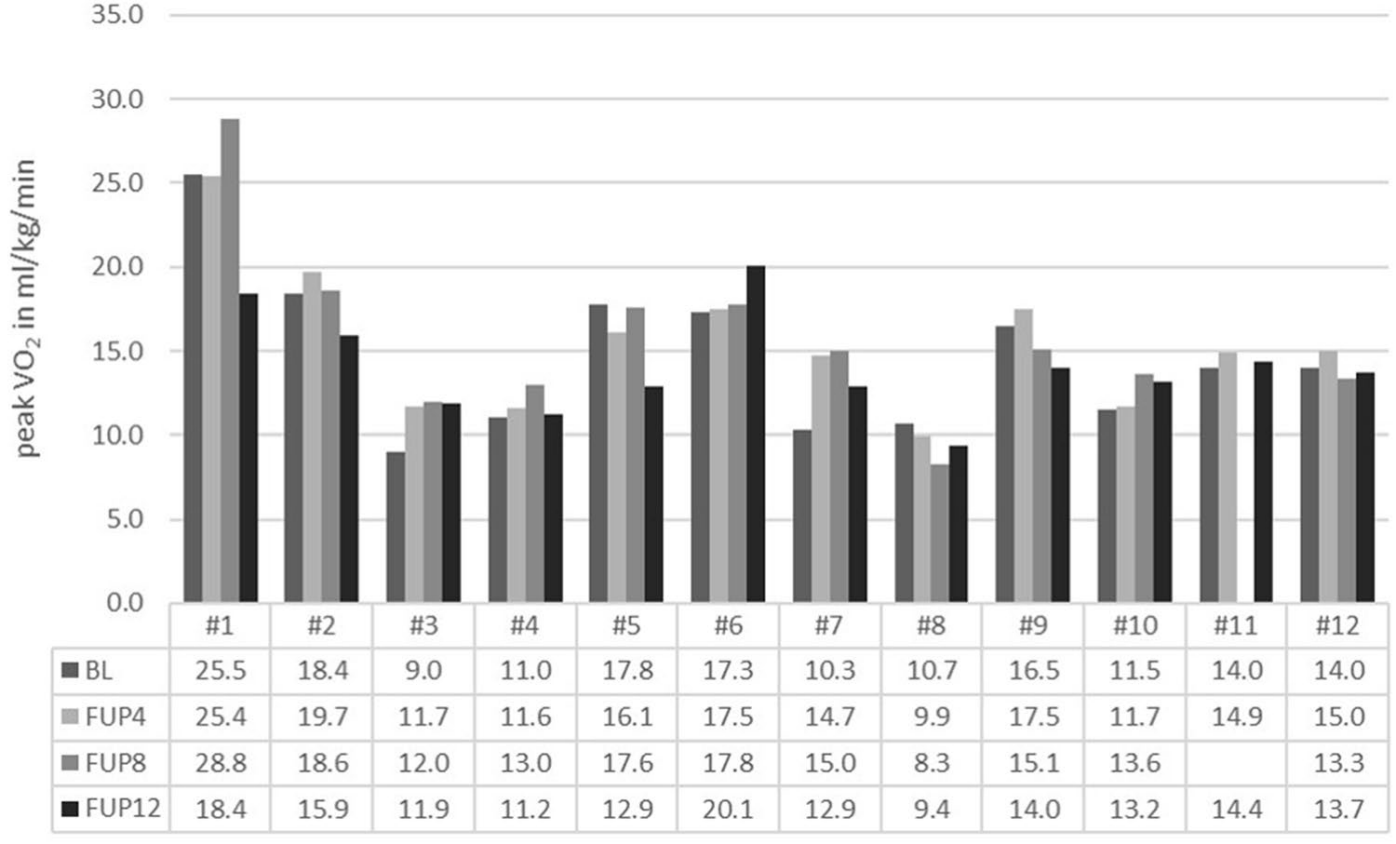
PeakVO_2_ of each patient at baseline and during follow-up after 4, 8 and 12 months. *BL* baseline, *FUP4* follow-up at 4 months, *FUP8* follow-up at 8 months, *FUP12* follow-up at 12 months.

**Figure 3:**
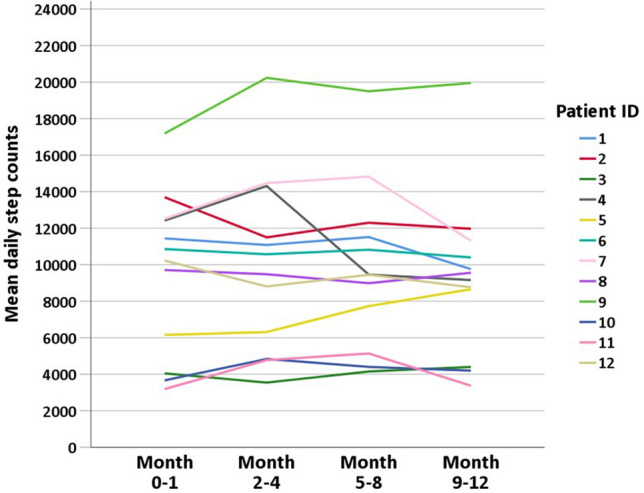
Daily step counts of each patient at baseline and during follow-up.

Exercise capacity was very heterogeneously distributed in the study sample at baseline, with median peakVO_2_ 14.0 ml/min/kg (quartiles 10.7, 17.7: for details refer to Fig. 2). During follow-up, peakVO2 and peakVO2% of predicted tended to rise after 4 months and 8 months, but this increase was not consistently observed after 12 months (p for trend for both = 0.03; Table 5). The other CPET parameters did not change substantially (best *p* = 0.10; Table 5). Walking distance in the 6-min walking test increased 2.2–4.4%, but not significantly (*p* = 0.11). Daily step count and activity time, did not change over time (Fig. 3 and Table 5; *p* = 0.11 and *p* = 0.22, respectively). The wrist-worn pedometer was well tolerated and worn in over 86% of days at least 1000 min.

## Discussion

The current non-randomized pilot study aimed to assess the safety, efficacy, and feasibility of an activity-monitored, physician-supported, dedicated training group for patients with heart failure. During a follow-up-time of 12 months, no training-associated events occurred; hospitalization rate was lower than the year prior to the study, and patients adhered reasonably well to the suggested time schedules (76% adherence to offered training sessions). Quantitative parameters measuring exercise capacity did not improve consistently, but amongst markers of wellbeing and heart failure severity some positive trends emerged regarding NT-proBNP levels, LVEF, and quality of life.

Physician-supported ambulatory training groups for patients with heart diseases, predominantly coronary artery disease, have a long tradition in Germany [1]. In contrast, patients with symptomatic heart failure were often excluded as their participation was considered unsafe, despite lacking data supporting such a notion, and despite opposite and strong recommendations from national and international guidelines [3, 21]. Only recently (January 2020) has this large patient group been specifically addressed in a position paper of the German Cardiac Society, which made a request to implement dedicated heart failure training groups in Germany. Meanwhile, a respective reimbursement scheme for heart failure training groups has been approved, which allow for the higher supervision efforts compared to conventional HSGs [6]. Due to the COVID-19 induced lockdown, uptake of heart failure training groups has been poor, thus experiences with and evidence arising from heart failure training groups are still sparse. The “HIP-in-Würzburg study” started in November 2018 before the position paper was published, and ended in March 2020. Thus, the study was largely unaffected by the pandemic regulations except for the last patient, who terminated the study 2 months earlier than intended.

Previous studies in inpatients and outpatients with heart failure showed multiple benefits of exercise on cardiovascular outcome, quality of life, rehospitalization rate and—in longer-term studies—even mortality [22–27]. Although the positive effects on hard clinical endpoints were modest and not consistently reproducible, no major negative effects of physical exercise in heart failure have yet became apparent, and benefits are thought to outweigh the risks by far [3, 21]. In our study, no patient died during follow-up, and the number of hospitalizations was lower than the year prior to study entry. Of course, due to the natural progression of the disease, rhythm disturbances, hospitalizations, and other cardiovascular events may occur independently from the exercise training and more frequently than in healthier heart conditions. Hence, they should not necessarily lead to a discontinuation of training sessions [28, 29]. Physician-guided training might be of even of higher importance in patients with advanced heart diseases than in healthier cardiovascular subjects attending the conventional HSGs [6].

There is a discussion about which training modality is superior for patients with heart failure [30]. According to current recommendations, we chose a combination of endurance and strength training, as this combination is supposed to increase exercise capacity most effectively in this patient group [31, 32]. Exercise capacity is an acknowledged indicator of better prognosis in heart failure; hence, it is a central surrogate of the success of training programs [33]. In our analysis, we did not observe meaningful and consistent improvements in exercise capacity, derived by CPET, 6MWT or the activity indices. However, our pilot study was not statistically powered to detect such changes. Further, the baseline cardiovascular fitness level assessed peakVO_2_ was very heterogeneous amongst participants, ranging from 9 ml/min/kg to up to 26 ml/min/kg. Although we intended to provide an individualized training program, the fitter patients might have benefitted from greater exercise intensity, duration and/or frequency of training sessions and therefore may have been under-challenged by our training settings. Thus, selection of patients according to their physical capacity applying a stress test rather than simplistically relying on ejection fraction or NYHA functional class might be key in ensuring less heterogenous group composition and therefore more efficient training results. An increase in LVEF has also been observed in other studies examining the effect of exercise training [34]. Of note, we observed a few relevant changes in heart failure medication as three patients were switched from ACEi/ARB to ARNI during follow-up and one patient received ivabradine additionally. Our study lacked the statistical power to judge on the associated consequences of the adjusted medication.

Benefits in quality of life have been frequently reported in training studies in heart failure [35], and could be partially observed in this pilot study. We found a significant increase in the KCCQ physical function and social limitation domain and respective trends for the overall and clinical summary scores. The observed increments of about 10 score points are clinically relevant [36], and underscore the potential of structured exercise programs in this vulnerable patient group.

## Limitations and strengths

The current pilot study was not powered, either in size or duration, to allow for reliable conclusions concerning the efficacy of heart failure training groups. However, we were able to evaluate the feasibility of weekly offered heart failure training groups in real life. In particular, positive secondary findings of such a regular schedule became apparent, including improved adherence and the optimization of guideline-recommended drug treatment. Further, patients in our study sample differed in several points from patients considered eligible for participation in heart failure training groups by the position paper (i.e., LVEF < 40%, NYHA functional class III, etc. [6].). The optimal criteria for group composition still need to be clarified. Further, equipping heart failure patients with a smartwatch including pedometer function might be an appealing method to track patients and motivate them to increase their individual daily activity. However, most patients with heart failure are elderly and not familiar with such advanced technology. Thus, proper handling of a smartwatch might have been an issue in our study, and a simple pedometer might have been more reliable.

## Conclusion

In conclusion, the current pilot study found that the concept of a dedicated heart failure training group was feasible, safe, and yielded improved quality of life and symptoms burden over a 12-months period. Participation in such training groups should be offered to all patients with heart failure who are not confident to participate in HSGs. Our experiences and considerations could be used to establish local heart failure training groups and will inform future trials.

## Data Availability

The submitted work is original and has not been published elsewhere in any form or language (neither partially nor in full).
